# Evidence of Waterborne Parasites in Mussels for Human Consumption Harvested from a Recreational and Highly Productive Bay

**DOI:** 10.3390/microorganisms13091971

**Published:** 2025-08-22

**Authors:** Pilar Suarez, Italo Fernandez, José Luís Alonso, Gladys Vidal

**Affiliations:** 1 Environmental Engineering & Biotechnology Group (GIBA-UDEC), Environmental Science Faculty & EULA-CHILE Center, Universidad de Concepción, Concepción 4070386, Chile; pilarsuarez@udec.cl; 2 Water Research Center for Agriculture and Mining (CRHIAM), ANID Fondap Center, Victoria 1295, Concepción 4070411, Chile; 3 Laboratorio de Parasitología, Facultad de Ciencias Biológicas, Universidad de Concepción, Concepción 4070386, Chile; itfernan@udec.cl; 4 Instituto de Ingeniería del Agua y Medio Ambiente, Universitat Politècnica de València, 46022 Valencia, Spain; jalonso@ihdr.upv.es

**Keywords:** waterborne, coastal fecal pollution, parasites, mussels

## Abstract

Coastal fecal contamination is a global public health concern, particularly due to waterborne protozoan parasites such as *Giardia duodenalis* and *Blastocystis* sp. Concepcion Bay (Chile) is an important recreational and productive area in Chile. Nevertheless, it is impacted by two submarine outfalls and a rural sewage treatment plant, which may contribute to fecal pollution. This study evaluated the presence of waterborne parasites in *Aulacomya atra* mussels intended for human consumption. The mussels were collected from three sectors of the bay: northern, central, and southern. A total of 600 mussels were analyzed as accumulators using PCR targeting SSU-rDNA and *β-giardin* genes for the detection of *Blastocystis* sp. and *G. duodenalis*, respectively. Additionally, thermotolerant coliforms were quantified using the most probable number (MPN) method. Both parasites and coliforms were detected in all sectors, with the southern zone showing the highest number of positive samples, indicating a localized public health concern. This is the first report of these protozoa in mussels from Concepción Bay. The findings highlight the need for regulatory frameworks to control protozoan discharge and reduce pathogen transmission risks in coastal ecosystems, especially in areas with high recreational and economic activity, both in Chile and worldwide.

## 1. Introduction

Several cities in Chile and around the world are in coastal zones, particularly bays, where high population densities present significant challenges for sewage management. In some bays, geomorphological constraints limit the feasibility of conventional sewage treatment plants. As a result, submarine outfalls are commonly employed as the primary sewage disposal technology [1,2,3,4]. However, submarine outfalls remain a controversial solution, as they primarily remove solids and oils from sewage without incorporating disinfection steps for pathogenic microorganisms [1].

Pathogen reduction in submarine outfall systems depends largely on environmental conditions such as dilution, ocean currents, salinity, seawater temperature, and solar radiation. These factors have been reported to eliminate up to 95% of fecal coliforms [1,5,6,7]. However, the effectiveness of pathogen removal is highly site-specific and susceptible to fluctuations in environmental parameters [8,9]. Under climate change scenarios, these variables may vary significantly, potentially compromising the reliability of natural pathogen attenuation mechanisms [10,11,12,13].

In Chile, microbial seawater quality near submarine outfalls is evaluated through the quantification of fecal coliforms [14]. Currently referred to as thermotolerant coliforms, this bacterial group serves as an indicator of fecal contamination and may be associated with a wide range of pathogens, including viruses and protozoan parasites. However, several studies have demonstrated that thermotolerant coliforms do not consistently correlate with the presence of other fecal-origin microorganisms, particularly protozoan pathogens [15,16,17,18].

Waterborne protozoan parasites represent a significant global public health concern, contributing to an estimated 1.7 billion cases of diarrhea and over 842,000 deaths annually [19]. Among the most prevalent species are *Cryptosporidium* sp. and *Giardia duodenalis*, while *Blastocystis* sp. has recently emerged as a relevant pathogen in outbreak scenarios [20]. These protozoa are classified as emerging contaminants due to their environmental persistence and potential to affect both human and animal health [21,22,23].

Their life cycle begins with an infected host shedding parasitic forms through feces [21]. Once released into the environment, these forms can contaminate water and food sources. Upon ingestion by a new host, the cysts traverse the digestive tract, differentiate into trophozoites in the intestine, and initiate infection. This process perpetuates the transmission cycle, as the newly infected host also excretes cysts [21]. Transmission is primarily linked to exposure to water contaminated with human or animal fecal matter. Epidemiological studies indicate that approximately 22% of protozoan outbreaks are associated with the ingestion of feces-contaminated drinking water [23,24].

Given the associated health risks, water quality monitoring has become increasingly important in public health strategies aimed at preventing waterborne parasitic diseases [20,25,26,27]. This is particularly critical in recreational areas such as swimming beaches, where approximately 75% of infections are reported to occur during aquatic activities [20]. Protozoan parasites possess highly resilient structures that enable them to persist in marine environments, especially within sediments, for extended periods [28]. These organisms can accumulate in commercially valuable seafood, particularly shellfish and mussels, posing a direct risk to consumers [29,30,31,32,33].

As filter-feeding organisms, mussels continuously extract suspended particles and nutrients from the surrounding water. *Aulacomya atra*, in particular, has been reported to filter up to 25 L of water per day [34,35]. Distributed throughout the benthic zone, *A. atra* is exposed to contaminants even in bays with strong hydrodynamics and high water renewal, facilitating the accumulation of pathogens and positioning it as a potential vehicle for transmission [31].

Concepción Bay (Chile) is one of the most ecologically productive marine zones globally, driven by the Humboldt Current System and seasonal upwelling that enhance nutrient availability [36]. Between 2022 and 2023, approximately 79,126 tons of wild *A. atra* mussels were harvested by artisanal fisheries, making it the second most consumed marine product in Chile [37]. However, *A. atra* has been identified as an accumulator of protozoan parasites, indicating potential parasitic contamination in the region [31,38].

Concepción Bay presents a unique case study for evaluating waterborne protozoan contamination due to the presence of two submarine outfalls and a rural sewage treatment facility located in distinct sectors of the bay. In Chile, waterborne parasites have been scarcely studied in aquatic environments. Therefore, this research represents one of the first studies focused on this aspect, particularly in the bay under investigation. It is hypothesized that filter-feeding organisms present in Concepción Bay accumulate waterborne parasites as a result of the bay functioning as a receiving body for various effluents. This study aims to determine the presence of *Giardia duodenalis* and *Blastocystis* sp. in *A. atra* mussels harvested for human consumption from recreational and commercially productive areas of the bay.

## 2. Materials and Methods

### 2.1. Description of Study Area

**Figure 1:**
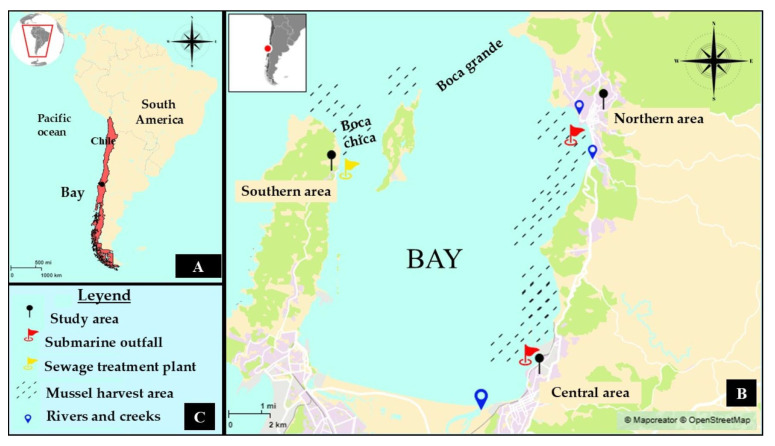
Location and characterization of study area (Mapcreator program). (**A**) Location on map of Bay of Concepción in South America. (**B**) Sampled areas and locations of mussel harvest areas, submarine outfalls, sewage treatment plant, and rivers and estuaries (red point indicate the location of the Bay in South America). (**C**) Map legend.

Concepción Bay, located in south-central Chile (36°40′ S, 73°02′ W), was selected as the study area due to two key attributes: (1) it receives effluent from a sewage treatment plant and two submarine outfalls, and (2) it supports both recreational activities and the extraction of marine products, particularly mussels (Figure 1). The bay is semi-enclosed, covering an area of approximately 170 km^2^, with an average depth of 18.5 m and a maximum depth of 45 m. Its estimated seawater volume is 3.1 × 10^9^ m^3^ [39].

The submarine outfalls are located approximately 1300 m offshore [40], while the sewage treatment plant discharges directly onto a beach in the southern sector of the bay. Additional freshwater inputs are provided by estuaries and rivers (Table 1). The bay is surrounded by both urban and rural settlements, with a combined population exceeding 45,000 inhabitants. The main economic activities in the region include artisanal fisheries and tourism, making it a socially and economically significant coastal zone. The areas of harvesting mussels are defined by Chilean regulation [37].

### 2.2. Mussel Sampling

The mussel used in this study was the cholga, identified taxonomically based on its morphological characteristics as *Aulacomya atra* [41]. A total of 600 fresh mussels from the northern, central, and southern areas (200 mussels per area) of the bay were purchased from local scuba divers when they arrived at the beach (Figure 1). Mussels were harvested during the spring (October and November 2023) to avoid water runoff and extreme rainfall events. It is also important to note that during the autumn and winter months (April to September), artisanal divers do not harvest mussels due to a fishing ban established by Chilean fisheries regulations [37]. They were transported on ice to the Parasitology Laboratory of the University of Concepción.

### 2.3. Parasite Cyst Purification

The mussels were grouped into 5 specimens to improve detection sensitivity. Each specimen was washed with sterile distilled water. Then, the valves were removed, and the hepatopancreas and gills were extracted. These organs were homogenized in a porcelain mortar with 15 mL of phosphate-buffered saline (PBS) and filtered through a strainer. The filtrate solution was centrifuged (10 min at 600 *g*, room temperature), and the pellets containing parasite cysts were collected and stored at 4 °C until cyst purification.

Cyst purification was performed using the sucrose flotation technique [42,43], with some modifications. The sucrose solution (20 mL of 0.85 M solution) was added to each of the tubes that contained the pellet. Then, the tube was centrifuged (600 *g* for 10 min at room temperature), and 7.5 mL was extracted from the surface and transferred to a clean centrifuge tube. This solution was dissolved in 7.5 mL of PBS, centrifuged (10 min at 600 *g*, room temperature), and 12 mL of the supernatant was removed, leaving the parasite cysts in the remaining volume (2 mL). Finally, all samples were stored at 4 °C until analysis.

### 2.4. DNA Extraction

DNA extraction consists of two lysis steps and DNA purification. The steps performed for the extraction are described below [44].

#### 2.4.1. Thermic Lysis

From the purified cyst sample (2 mL), 1 mL was transferred to an Eppendorf tube and subjected to thermal rupture through cold–heat cycles. Ten cycles of 10 min at −41 °C and 10 min at 95 °C were performed on each sample. Then, these thermally treated samples were subject to enzymatic lysis.

#### 2.4.2. Enzymatic Lysis

Following the thermal lysis, the samples were subjected to enzymatic lysis using the proteinase K enzyme (EO0491, Thermofisher, Waltham, MA, USA). For a 1 mL sample, 200 µL of lysis buffer (100 mM Tris-HCl pH 7.5, 0.5 M EDTA, 10% SDS, and proteinase K) was added. The samples were incubated for 5 h at 56 °C. Then, the samples were stored at −20 °C until genomic DNA purification.

#### 2.4.3. Genomic DNA Purification

The genomic DNA was purified using the commercial QIAGEN DNEASY Power Soil^®^ Pro kit (Cat. 47014, Thermofisher). This step was carried out according to the provider protocol. The genomic DNA was quantified using the TECAN Infinitive^®^ 200pro instrument (Männedorf, Switzerland) with Control Infinitive Reader software (v.1.9). The samples were stored at −40 °C until PCR assay.

### 2.5. Parasite Gene Identification

The two waterborne parasites in the mussel samples were detected by conventional PCR, indicating the presence of their DNA. For this, the following genes were amplified: SSU-rDNA (forward primer: 5′-GGA GGT AGT GAC AAT AAATC-3′; and reverse primer: 5′-TGC TTT CGC ACT TGT TCATC-3′) and *β-giardin* (forward primer: 5′-AAGCCCGACGACCTCACCCGCAGTGC-3′; and reverse primer: 5′-GAGGCCGCCCTGGATCTTCGAGACGAC-3′) for *Blastocystis* sp. and *G. duodenalis*, respectively [45,46]. For the PCR, a Dream Taq green polymerase (K1081, Thermofisher) was used. The PCR conditions were as follows: 95 °C for 4 min; 40 cycles of 95 °C for 30 s, 54 °C for 30 s, and 72 °C for 30 s; and a final extension of 72 °C for 5 min. For positive control, 1 mL of two human fecal samples was used, each containing *Blastocystis* sp. and *G. duodenalis* and previously examined by microscopy. These samples were available at the Parasitology Laboratory of the University of Concepción (Chile) and had already been analyzed by PCR [38]. Both samples were processed using the same procedures applied to the mussel-derived specimens. For the negative PCR control, DNase-free water was used instead of DNA in order to rule out potential contamination. The PCR products were subject to agarose (2%) gel electrophoresis and visualized with the Safeview Plus staining reactive (FER00SV500UL; Fermelo Biotec, Santiago, Chile).

### 2.6. Thermotolerant Coliform Determination

The microbiological quality of the mussels was determined using the thermotolerant coliform estimation method. For this, fresh mussels (100 g) from each study area were used in triplicate. The PBS solution (15 mL) was added to the mussels and homogenized in the BagMixer homogenizer for 1 min at a speed of 8 strokes/second (BagMixer400^®^, Interscience, Saint-Nom-la-Bretecche, France). This homogenate was subject to serial dilution in tubes containing 10 mL of lauryl sulfate lactose medium and incubated at 35 °C for 18 h in aerobiosis, as described in standard method protocols [47]. The positive tubes were inoculated with 10 mL of Escherichia Coli medium (EC) for 24 h at 44 °C in aerobiosis. The result was expressed as the most probable number per 100 g of fresh mussels (MPN 100 g^−1^ mussels).

### 2.7. Statistical Analysis

Statistical analysis of the data was carried out in the GraphPad Prism program version 10.0.2. The mean and standard deviation were calculated. Mean comparison was performed via one-way ANOVA with a significance level of 0.05 after normal distribution and homogeneity verification. Then, a multi-comparison test was performed to determine the significant variation between samples (Tukey’s Test).

## 3. Results

### 3.1. The Determination of Waterborne Parasites in Mussels Harvested from the Bay

Mussels have a high commercial value on this bay and have been proposed as microorganism accumulators to study contamination in the marine environment. Therefore, the presence of *G. duodenalis* and *Blastocystis* sp. was evaluated in mussels. The results are shown in Table 2.

The most frequently identified parasite was *G. duodenalis*. This parasite was found in 30 groups (25%) out of a total of 120 groups analyzed. *Giardia duodenalis* was predominantly detected in the northern and southern sectors of the bay, with 14 sample groups (35%) and 13 sample groups (32.5%) testing positive, respectively. In the central area, it was only identified in three (7.5%) samples.

*Blastocystis* sp. was detected in 22 sample groups (18%) out of a total of 120 analyzed. The highest prevalence was observed in the southern sector of the bay, with 13 positive sample groups (32.5%). In contrast, the northern and central sectors exhibited lower detection rates, with five (12.5%) and four (10%) positive sample groups, respectively. Overall, the southern area registered the highest presence of the three parasites evaluated. This zone houses a sewage treatment plant and is situated opposite an island, while both the northern and central zones contain submarine outfalls (Figure 1), which may contribute to the observed contamination levels.

### 3.2. The Determination of Thermotolerant Coliforms in the Mussels Harvested from the Bay

**Figure 2:**
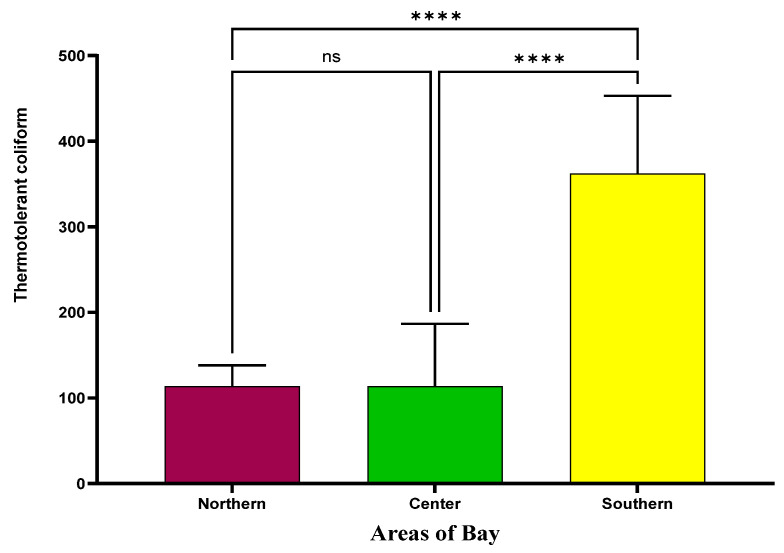
Thermotolerant coliform analysis of mussels harvested from the bay (GraphPad Prism). Notes: (ns) not statistically significant; (****): statistically significant difference between the means (*p* < 0.0001).

The presence of thermotolerant coliforms in mussels reflects fecal contamination in the marine environment, attributable to their accumulation capacity. Analysis was conducted across three sectors of the bay, revealing a significantly higher concentration of coliforms in the southern area (Figure 2). Specifically, the mean value in this sector was 4.6 × 10^2^ MPN per 100 g^−1^, markedly exceeding the levels observed in the northern and central areas, which both averaged 1.1 × 10^2^ MPN per 100 g^−1^ and showed no statistically significant difference between them.

## 4. Discussion

The presence of fecal pathogens in seawater is a public health risk, especially in recreational and productive areas around the world. Concepción Bay, a biologically rich and economically vital coastal ecosystem in Chile, represents an interesting study area due to anthropogenic pressures, particularly fecal contamination. This study provides molecular evidence of *G. duodenalis* and *Blastocystis* sp. in *A. atra* mussels, reinforcing the role of filter-feeding bivalves as effective bioindicators of waterborne protozoan pollution [17,21,31,32,46].

The spatial distribution of protozoan DNA and thermotolerant coliforms suggests possible fecal contamination, with the southern sector exhibiting the highest microbial load. The geomorphology and hydrodynamics of the bay could affect water renewal and promote parasite sedimentation [48,49]. These phenomena may enhance parasite exposure to mussels by promoting the continuous resuspension of sediment within the water column, driven mainly by anthropogenic disturbances and upwelling events [50,51].

The fecal pression produced by the submarine outfalls and sewage treatment plants in this bay could generate continuous fecal contamination. Moreover, this bay contains households that are not connected to a sewage system, explaining the presence of these parasites and thermotolerant coliforms [52,53,54].

The detection of *Blastocystis* ST3 and *Cryptosporidium parvum* in mussels and treated sewage, and Hepatitis A virus and thermotolerant coliforms in seawater, has been observed in this bay [10,13,38,55,56].

Although no protozoan-related outbreaks have been documented in Chile, the widespread consumption of raw mussels, particularly *A. atra*, presents a latent public health risk [38]. The asymptomatic nature and delayed onset of protozoan infections complicate the epidemiological association to foodborne transmission routes.

These findings highlight the need to expand current monitoring frameworks beyond bacterial indicators to include emerging protozoan contaminants in seawater quality regulations. Integrating molecular diagnostics into routine surveillance could enhance early detection and risk assessment. Future research should focus on evaluating parasite viability, genotype or subtype identification, and assessing the performance of sewage treatment systems under unstable environmental conditions.

## 5. Conclusions

Based on the results of this study, it is concluded that *Blastocystis* sp. and *G. duodenalis* waterborne parasites are present in mussels harvested from different areas of Concepción Bay (Chile). The waterborne parasite presence varies according to the different areas of the bay, which could be related to the presence of two submarine outfall and a rural sewage treatment plant. It is important to re-evaluate the external environmental factors (temperature, dilution factors, tidal effect, among others) that could influence the capacity of pathogen elimination of submarine outfalls in this bay. This is the first report of these protozoa in mussels from Concepción Bay. Future research should focus on evaluating parasite viability, genotype or subtype identification, and assessing the performance of sewage treatment systems under unstable environmental conditions. Finally, it is essential to enhance the quality assurance of microbial detection in seafood and seawater, particularly for parasites that are environmentally persistent and resistant to disinfection treatments.

## Figures and Tables

**Table 1:** The characteristics of the different parts of the study area.

Area	Population ^1^	Sewage Discharge ^2^	Maximum Daily Flow Rate (m^3^ day^−1^)	Population Served by Plant	Other Water Discharge
Northern	54,946	Submarine outfalls	19,785 ^2^	54,946	Collen and Bellavista creeks
Center	46,176	Submarine outfalls	35,554 ^2^	46,176	Lirquen Creek and Andalien river
Southern	92,843	Sewage Treatment Plant	30,000 ^3^	200 ^4^	Raw sewage from houses

Notes: (^1^) Data from Chile 2017 census; (^2^) data from SSIS; (^3^) data calculated by estimating the production of 150 L day^−1^ of sewage per person in a rural zone; (^4^) people who live in the south but do not use the sewage treatment plant.

**Table 2:** Parasites detected in mussels from different areas of the bay.

Area	Total Mussel Harvested	Mussel Sample *	*Giardia duodenalis*	*Blastocystis* sp.
Northern	200	40	14 (35 **)	5 (12.5 **)
Center	200	40	3 (7.5 **)	4 (10 **)
Southern	200	40	13 (32.5 **)	13 (32.5 **)
Total	600	120	30 (25 ***)	22 (18 ***)

Notes: (*) Mussel sample: group of five mussels from total mussels for each area; (**) percentage obtained considering samples per area (40); (***) percentage obtained considering total number of samples (120).

## Data Availability

The original contributions presented in this study are included in the article. Further inquiries can be directed to the corresponding author.
